# Genetic Affinity of the Bhil, Kol and Gond Mentioned in Epic *Ramayana*

**DOI:** 10.1371/journal.pone.0127655

**Published:** 2015-06-10

**Authors:** Gyaneshwer Chaubey, Anurag Kadian, Saroj Bala, Vadlamudi Raghavendra Rao

**Affiliations:** 1 Estonian Biocentre, Riia23, Tartu 51010, Estonia; 2 5 Ror Colony, Behind Sector 7, Karnal, Haryana132001, India; 3 Institute of Scientific Research on Vedas, I-SERVE Delhi Chapter, C-6 / 302, Clarion the Legend, Gurgaon 122011, India; 4 Department of Anthropology, University of Delhi, North Campus, Delhi 110007, India; Centre for Cellular and Molecular Biology, INDIA

## Abstract

Kol, Bhil and Gond are some of the ancient tribal populations known from the *Ramayana*, one of the Great epics of India. Though there have been studies about their affinity based on classical and haploid genetic markers, the molecular insights of their relationship with other tribal and caste populations of extant India is expected to give more clarity about the the question of continuity *vs*. discontinuity. In this study, we scanned >97,000 of single nucleotide polymorphisms among three major ancient tribes mentioned in Ramayana, namely Bhil, Kol and Gond. The results obtained were then compared at inter and intra population levels with neighboring and other world populations. Using various statistical methods, our analysis suggested that the genetic architecture of these tribes (Kol and Gond) was largely similar to their surrounding tribal and caste populations, while Bhil showed closer affinity with Dravidian and Austroasiatic (Munda) speaking tribes. The haplotype based analysis revealed a massive amount of genome sharing among Bhil, Kol, Gond and with other ethnic groups of South Asian descent. On the basis of genetic component sharing among different populations, we anticipate their primary founding over the indigenous Ancestral South Indian (ASI) component has prevailed in the genepool over the last several thousand years.

## Introduction

Knowledge about the past comes through different disciplines where researchers look at history through different lenses. And in many cases, these interdisciplinary studies land on the same conclusions [1,2]. However, in case of India, investigations from different disciplines have historically been highly contrasting [3,4]. India, also known as a ‘land of spiritual heritage’, has a deep history of civilisation, which is embedded in to multiple oral, traditional and written records. Much of this knowledge is rooted in oldest scriptures, the *Vedas*, which are four in number, namely *Rigveda*, *Yajurveda*, *Samaveda and Atharvaveda*. Then, there are *Puranas*, *Upanishads*, *Brahmanas and Aranyakas*, of which *Vedas* are said to be the precursors [5]. There is no consensus among historians regarding the date of compilation of the *Vedas* as well as the historical dates for the various *Puranas*, *Upanishads* and epics [6–10]. A comperative analysis of such mythological sources may provide a concencus about the structuring of the ancient societies and rituals. More recently, some scholars have provided strong evidence about the chronology of these events hinting at a deep-rooted civilization, developing indigenously for over several thousand years [8,11–15].

Our survey on mythological sources has revealed detailed information about the ancient Indian society structure as well as relations of different tribal and caste groups and their rituals [10,11,16–18]. In many of these literary sources, names of various castes and tribal groups have been mentioned, including those of several surviving tribal groups (e.g. Bhil or Bheel, Kol, Gond, Savara, Oraon, Kirata, Ahirs, Nagas etc) [17–23]. It is already evident that during the *Ramayana* era, Indian society was well-stratified [16,17,21,23–26]. The Bhil, Kol and Gond are three major Indian tribes that have been widely acknowledged in the epic *Ramayana*, particularly in the portions known as the *Ayodhyakanda*, *Aranyakanda and Kishkindhakanda* [19,20,22–27]. It should be emphasised here that, Gond and Bhil are the top two tribal populations of modern India in terms of population size [28].

The Bhils are primarily from Central India and speak the Bhil language [28]. They have significant presence in states of Gujarat, Madhya Pradesh, Chhattisgarh, Maharashtra and Rajasthan as well as in the northeastern state of Tripura. Bhils are further divided into a number of endogamous territorial divisions, which in turn have a number of clans and lineages [22]. The Kol tribe in Uttar Pradesh is found mainly in the districts of Mirzapur, Varanasi, Banda and Allahabad [28]. It is the largest tribe found in the state Uttar Pradesh. They are said to have migrated from Central India some five centuries ago [28]. The Kol are further divided into a number of exogamous clans, such as the Rojaboria, Rautia, Thakuria, Monasi, Chero and Barawire. The Gond people are spread over the states of Madhya Pradesh, eastern Maharashtra (Vidarbha), Chhattisgarh, Uttar Pradesh and Telangana [28]. With over four million people, they are the largest tribe in Central India. They speak the Gondi language, which is related to present Dravidian language family [28,29].

More than 25 years of genetic research on Indian tribal and caste populations involving classical markers to mtDNA/Y chromosome and more recently autosomes, have indicated complex demographic history of the subcontinent [3,30–39]. Alongwith debate over initial peopling of the subcontinent, the major hot topic now shifted towards the population expansion and admixture during and after Neolithic times [37–40]. However, large number of individuals as well as genetic markers are required to reach any firm conclusions. Thus, the strict endogamy and social structure make South Asia much more complex, unlike to Europe, where genetic analysis of a population can predict the genetic structure of immediate neighbor with some confidence. In recent years, there has been an increase in the number of in-depth genetic studies focussing on the genetic structure of the populations of India [35,37,40–48], but none of them have related specific tribal populations mentioned in the traditional literatures.

Therefore, in the present study, we make an attempt to evaluate two schools of thought emerging from the current scenario. The first school suggests that the tribal people are the aboriginal inhabitants, while the later migrants, i.e., the Dravidians followed by the Aryans have pushed them back in to small pockets in South India [49–52]. According to this school, the caste system was established by the aforementioned later migrants [11,50,52,53]. The alternative hypothesis advocates that all the caste and tribal populations of India have Paleolithic roots and share a common origin [3,15,33,54–60]. The differentiation observed in modern South Asian populations is mainly derived by strict endogamy, long term isolation and several evolutionary forces. More specifically, relying on each other, first, we seek to investigate the continuity *vs*. discontinuity of the genetic thread connecting the different populations of India. Second, keeping in mind the pivotal information extracted from *Ramayana*, we look specifically into the question: whether and to what extent the three major tribes (Bhil, Kol and Gond) share their genetic ancestry among them as well as with other contemporary caste and tribal populations?

## Material and Methods

This study was performed using control samples collected, genotyped and published for various population studies conducted in the last few years (S1 Table) [37–39,46,61–63]. All the ethical guideline have been followed. The tribal and caste populations grouped according to their language group. We grouped populations in to “Transitional” who have known information of language change in recent time [64,65]. A check for closely related individuals was carried out within each population study by calculating average *identity by state* (IBS) scores for all pairs of individuals [66]. We used PLINK 1.07 [66] in order to filter our dataset to include only SNPs on the 22 autosomal chromosomes with minor allele frequency >1% and genotyping success >99%. As background linkage disequilibrium (LD) can affect both PCA [67] and ADMIXTURE [68], we thinned the dataset by removing one SNP of any pair, in strong LD r2>0.4, in a window of 200 SNPs (sliding the window by 25 SNPs at a time).

We performed PC analysis using *smartpca* programme (with default settings) of the EIGENSOFT package [67] in order to capture genetic variability described by the first 5 components. The fraction of the total variation described by a PC is the ratio of its eigenvalue to the sum of all eigenvalues. In the final settings, we ran Admixture with a random seed number generator on the LD-pruned dataset twenty-five times at K = 2 to K = 12. Since the top values of the resulting log-likelihood scores were stable (virtually identical) within the runs of each K from K = 2 to K = 10, we can claim that convergence at global maximum was achieved. Thus, we omitted runs at K = 11 to K = 12 from further analysis.

Mean pairwise differences between different population groups were computed using *F*st distance measure by following the methods as described by Cockerham and Weir [69], Phylip [70] and MEGA [71] were used to construct the tree. The Plink software [66] was used to calculate the genetic diversity and to find the 25 nearest-neighbours for the Bhil, Kol and Gond individuals. To investigate the derived allele sharing of Bhil, Kol and Gond with the Eurasian populations, we computed *f3* statistics [37], taking African as an outgroup. For haplotype-based analysis (fineSTRUCTURE) [72], we made two different runs—first by taking all the Eurasian populations and second exclusively on the Central Asian, Pakistani and Indian populations. For the fineSTRUCTURE analysis, first samples were phased with Beagle 3.3.2 [73]. A coancestry matrix was constructed using ChromoPainter [72], fineSTRUCTURE was used to perform an MCMC iteration using 10000000 burning runtime and 10000 MCMC samples. A tree was built using fineSTRUCTURE with the default settings. All these information are plotted for the Bhil, Kol and Gond as a recipient of number of chunks from one another as well as from other ethnic group.

## Results and Discussion

**Fig 1:**
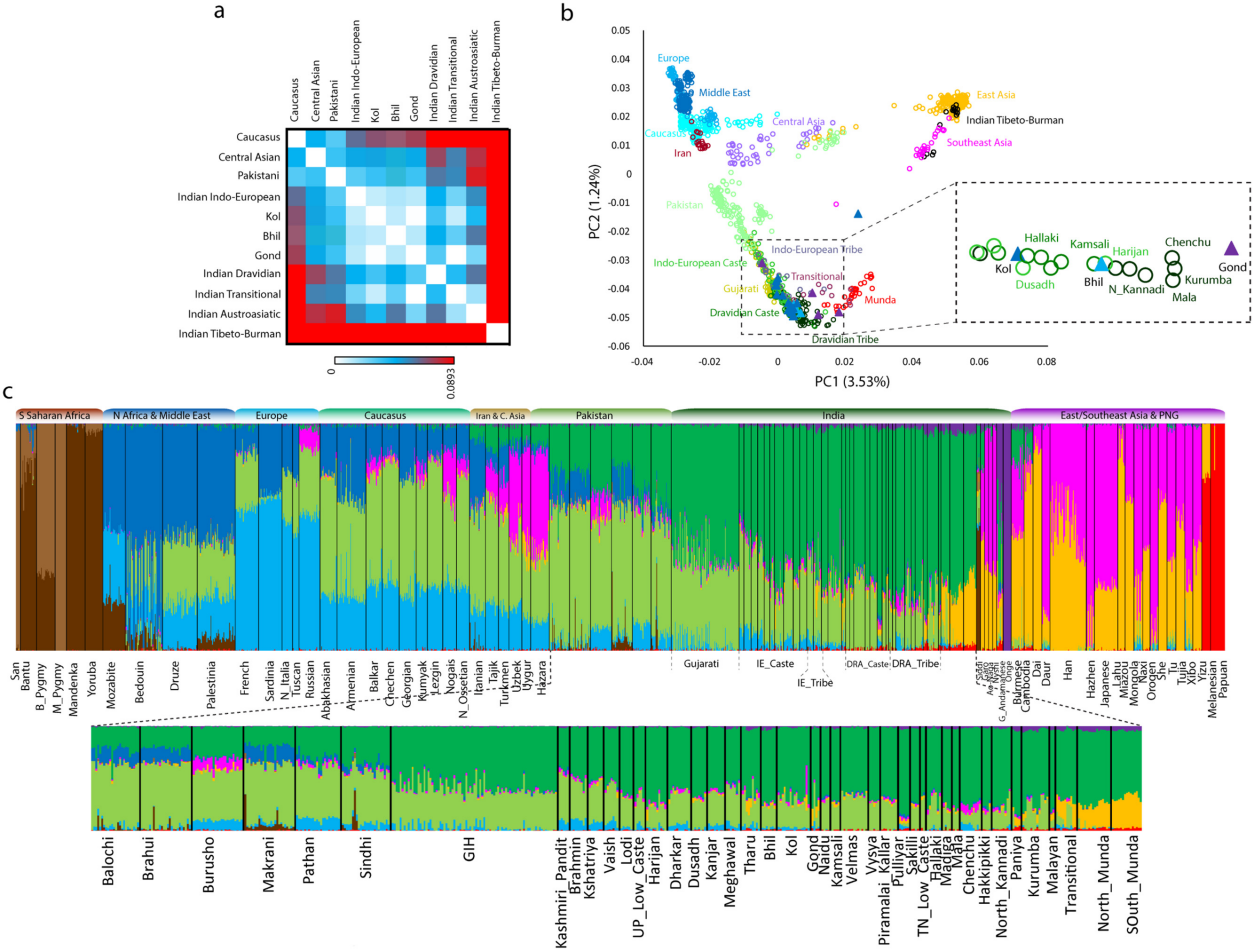
**a)** Regionwise population differentiation (*F*st) analysis of Bhil, Kol Gond with the Indian and other regional populations **b)** PCA (Principle Component Analysis) of Eurasian populations showing the placement of Bhil, Kol and Gond populations over the South Asian cline; the mean value of populationwise clustering of Bhil, Kol and Gond is zoomed-in inset figure. IE- Indo-European, DRA- Dravidian **c)** Individualwise ancestry proportion analysis inferred from ADMIXTURE representing ten ancestral populations of the world (K = 10).

We combined hundreds of thousands of autosomal markers generated from different studies (S1 Table) [37–39,46,61–63] and specifically looked into the population structure of Indian groups mentioned in classical literature. To find out the population clustering, we first ran the *F*st (population differentiation) algorithm [69] and drew a tree [70,71], rooting out the African populations (S1 Fig). All the Indian populations, except the present Tibeto-Burman speaking populations, are well separated from other continental populations and form a major cluster comprising present populations speaking Indo-European, Dravidian and Austroasiatic (Munda) languages (S1 Fig). The Pakistani populations are scattered in different clusters, where few of them (Sindhi, Pathan and Burusho) cluster loosely with Indians; Hazaras show an affinity toward Central Asians, and Balochi, Brahui and Makrani confirm an intermediate position because of shared recent African ancestry and gene flow [38,74,75]. The Bhil, Kol and Gond showed a closer affinity among them as well as with the extent Indo-European, Transitional and Munda speaking populations (Fig 1a and S1 Fig).

**Fig 2:**
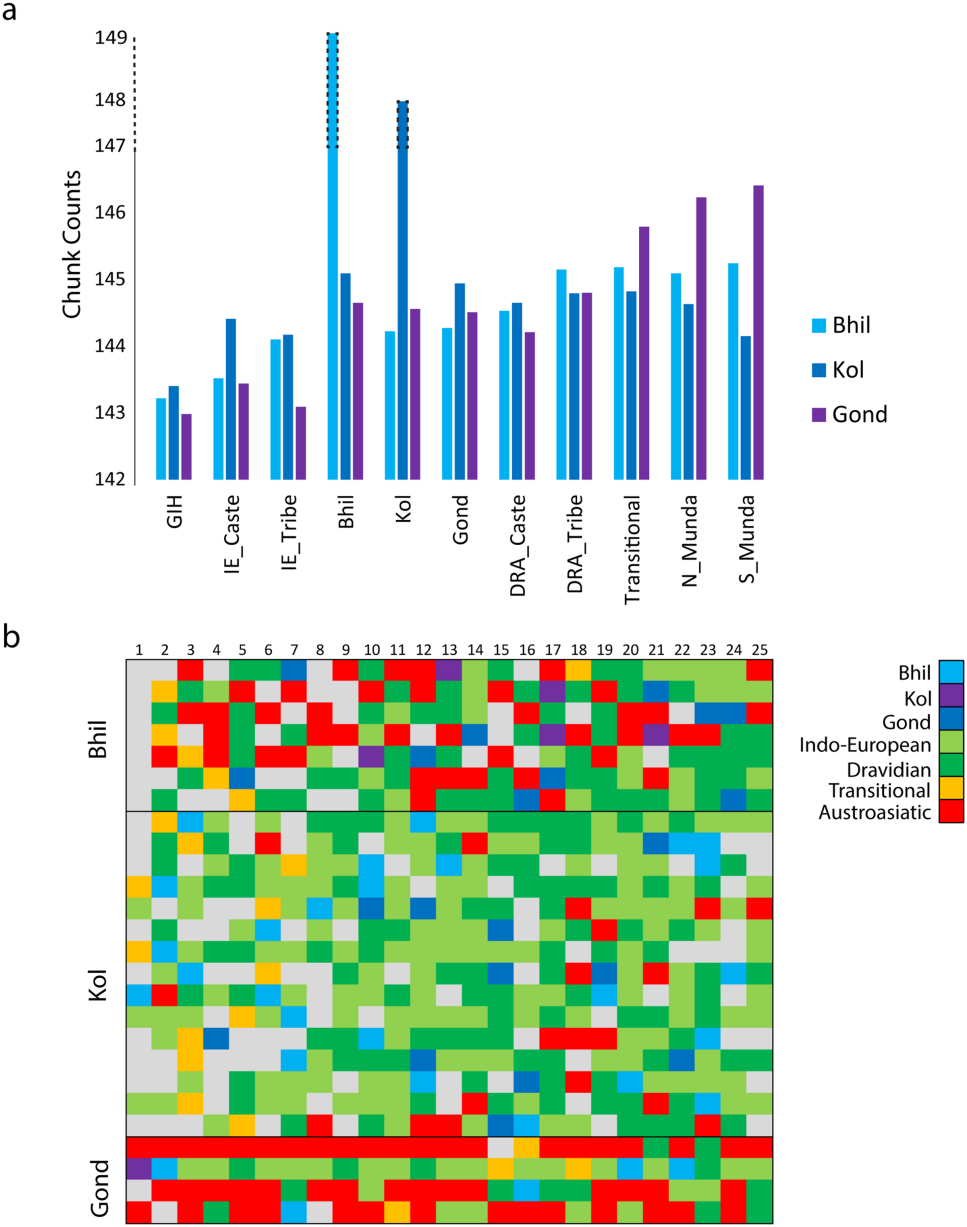
**a)** The number of chunks donated at inter and intra populations level for the Bhil, Kol and Gond with respect to the Indian, Central Asian and the Pakistani populations. **b)** Plot of 25 nearest neighbors of Bhil, Kol and Gond individuals. The match population individuals are colored in grey.

To get more deeper insight, we have used PCA (principle component analysis)[67] and ADMIXTURE [68], analysis using the same parameters as in our previous studies [38,39,45]. These analyses strengthened the inferences drawn from the *F*st analysis. The PCA on Eurasians placed Indian populations between East and West Eurasia (Fig 2a). The cline of Indian subcontinent ranges from Pakistani populations (closer to West Eurasians) to Indian Munda groups (closer to East Eurasians). Departing from its geographical position, Bhil was clustering together with Scheduled castes and Scheduled tribe populations of Uttar Pradesh (Harijan), Andhra Pradesh (Kamsali) and Karnataka (North Kannadi) states. Kol is joined with the neighbouring populations alongwith the Indian-cline, while Gond was deflating away from the Indian cline by uniting with the Munda speakers (Fig 1b). Further, we assessed the proportion of individual-wise ancestry drawn from a given number of inferred populations (K) using a maximum-likelihood based approach implemented in ADMIXTURE.

Consistent with previous observations [37,38], the South Asian populations’ genome are mainly made-up of two major components, which are distributed across the length and breadth of the subcontinent (Fig 1c). Alongnwith these two major components, there are four minor componets over the periphery of the subcontinent—the European and the Middle eastern components can be seen in Pakistani and northwest Indian populations, whilest the East/Southeast Asian components are present in nearby Munda and Tibeto-Burman speakers. (Fig 1c). The geographical distribution of the dark green component (ASI or Ancestral South Indian- unique to the subcontinent) was largely limited to the Indian subcontinent, and can be seen among all the populations of the subcontinent albeit in variable amount, whereas the second major component (light green: ANI or Ancestral North Indian (now ANE- Ancestral North Eurasian [76])) was shared with Central Asia, the Caucasus, Middle East and Europe (Fig 1c). The geographical origin of light green component (ANI or ANE) is so far unclear and more research is needed from unsampled area as well as from ancient DNA; however, the time of spread of this component from its origin place (either of any; the Caucasus, Near East, Indus Valley, or Central Asia) has happened more than 12.5 thousand years before [38], which is significantly earlier than the purported expansion of Dravidians and Aryans languages from outside the subcontinent. Notably, the Andaman Islanders are not the only population carrying the ASI component exclusively, as was suggested before [37]. Austroasiatic speakers (more precisely, the South Munda) of the subcontinent also seem to possess the ASI component in near unadulterated form (Fig 1c). More research with complete genome analysis would be required to clear the geographic center of the ANE component; however, it is evident from the present analysis that the dark green component (ASI) can be considered as a connecting thread for all the Indian populations (Fig 1c). Taken together, these results support the second hypothesis suggesting that all Indians, irrespective of their caste or tribal affiliations, share a common genetic ancestry, which is undoubtedly founded over the indigenous ASI component.

Our second question revolved around the three tribal populations mentioned in the ancient epic, their genome composition and affiliation with the surrounding caste and tribal populations. Based on information from *Ramayana*, we have considered these tribal populations to be ancient inhabitants of India, surviving from the times of the Stone Age [19,23]. If we assume that their genome carry the signature of peopling of ancient time, the assessment of their genomes and comparison with modern populations would test the scenario of continuity *vs*. discontinuity of prehistoric heritage. In case of continuity, we should see largely similar genome composition among contemporary caste and tribal populations of modern India. On the other hand, in case of discontinuity, these tribal populations should show a unique genome composition or they should emerge as an outliers in our cluster based analysis. Our extended analysis on *F*st, PCA and ADMIXTURE showed similar genome composition of these tribal populations, carrying both the ANE and ASI components (Fig 1a–1c, Table 1 and S1 Fig). We also calculated the genetic diversity of these populations with their neighbours (Table 2). The diversity of Kol, Bhil and Gond didn’t show any significant deviation from their neighbouring extent Indo-European, Dravidian and Munda speaking groups.

**Table 1:** The ANE and ASI admixture proportions of Bhil, Kol and Gond with respect to other South Asian groups.

Ethnic group	ANE	ASI
Pakistan	49.49 ± 10.88	23.78 ± 9.08
Bhil	19.81 ± 0.97	70.17 ± 1.13
Kol	25.06 ± 5.35	64.64 ± 9.84
Gond	15.55 ± 15.47	67.12 ± 11.71
Indian IE	33.57 ± 6.83	57.77 ± 8.29
Indian DRA	20.55 ± 9.6	70.08 ± 6.30
Transitional	9.88 ± 8.81	66.59 ± 2.92
Indian AA	0.69 ± 2.06	66.97 ± 4.94

IE—Indo-European, DRA—Dravidian, AA—Austroasiatic (Munda)

**Table 2:** The genetic diversity calculations of Bhil, Kol and Gond with respect to other South Asian groups.

Ethnic group	Genetic Diversity % (1-iBS) ± SD
Pakistan	26.89 ± 0.38
Bhil	25.26 ± 0.15
Kol	25.69 ± 0.32
Gond	25.66 ± 0.43
Indian IE	26.09 ± 0.34
Indian DRA	26.14 ± 0.47
Indian AA	25.23 ± 0.36

IE—Indo-European, DRA—Dravidian, AA—Austroasiatic (Munda)

The shared drift statistics analysis (*f3*) suggested that most of the derived alleles of Bhil, Kol and Gond are overwhelmingly shared with Indian caste and tribal populations (S2 Fig). Gond, Dravidian tribes and Austroasiatic (Munda) groups shared the highest derived allele with Bhils. Indo-European castes, Gond and Dravidian tribes were closest with Kol. Whereas, Munda, transitional and Dravidian tribal groups shared the peak derived alleles with Gonds.

The haplotype based fineSTRUCTURE [72] analysis showed that the studied populations (Bhil, Kol and Gond) received nearly all of their chunks from the Indian closeby populations (Fig 2a and S3 Fig). Leaving out the number of chunks coming from the same population, chunk donors for Bhil and Kol were coming from all the major Indian ethnic groups, while for the Gond, Indian Transitional and Munda groups were the major chunk donors. More specifically the haplotype based sharing analysis is in congruent with the *f3* statistics. The fineSTRUCTURE clustering analysis revealed 37 clusters when we have included Iranian, Central Asian, Pakistani, Indian and Cambodian populations (S4 Fig). Most of the Indian populations unite in Indian specific clusters except Kashmiri Pandits and few Gujarati individuals who fell together with the Sindhi and Pathan individuals in Pakistani specific clade. Our targeted populations are dispersed in various clades. All the Bhil individuals form a tight cluster with the individuals mainly from Dravidian caste, few Indo-European and Transitional individuals. Most of the Kol and Gond individuals show a higher level of variation by falling in to distinct clusters. To make an individual-wise comparison, we plotted top twenty five closest neighbours of studied populations (Fig 2b). It was expected that any population members would be closest to themselves and thereafter to members of other populations, which was also pertinent in the present study. Consistent with the above observations drawn from *F*st, PCA, ADMIXTURE, *f3* statistics and fineSTRUCTURE, there is no signature of large scale population replacement in the Indian subcontinent.

In conclusion, our high resolution analysis portraying the three ancient tribal populations, strongly rejects any incoming genetic signal of large scale recent (during the post-Neolithic) migration either of the present Dravidian or the Indo-European speaking populations to the subcontinent. We also concluded that the Indian populations preserve strong genetic signatures in support of a common ancestry. The studied tribal populations do share large number of genome among theselves as well as from o caste and tribal poulations. Notebly, the placement of various populations along the Indian cline is not solely governed by the geography, but also by the caste-tribe interaction and various other selectional forces. These patterns point to a complex demographic history of the subcontinent which has been shaped in-situ by admixture events at different time scale, as well as by intricate geographical heterogeneity and long term effect of several evolutionary forces.

## Supporting Information

S1 Fig Neighbour Joining (NJ) tree world populations inferred from *F*st distances of genomewide data. In the inset, the heatmap showing the inter and intra regional genetic affinity of the three tribal populations under investigation.
(TIF)

S2 Fig The plot of shared drift obtained by the *f3* = (Yoruba; Bhil/Kol/Gond, X). The *f3* values are plotted on Y axis against the X- targeted populations on X axis. C_Asia- Central Asia, IN_IE_Caste- Indian Indo-European Caste, IN_IE_Tribe—Indian Indo-European Tribe, IN_DRA_Caste- Indian Dravidian Caste, IN_DRA_Tribe—Indian Dravidian Tribe, IN_AA- Indian Austroasiatic (Munda).
(TIF)

S3 Fig Co-ancestry matrix plotted from fineSTRUTCURE analysis, showing the chunks donated by other Eurasian populations to the Bhil, Kol and Gond populations. (TIF)

S4 Fig Placement of Kol, Bhil and Gond individuals over the 37 clades obtained from the fineSTRUCTURE analysis. (TIF)

S1 Table The details of the populations (number of individuals and number of SNPs, used in the present study. (XLSX)
