# Correction: Genetic Affinity of the Bhil, Kol and Gond Mentioned in Epic *Ramayana*

**DOI:** 10.1371/journal.pone.0134200

**Published:** 2015-07-24

**Authors:** The PLOS ONE Staff

There are errors in the Funding section. The publisher apologizes for the error. The correct funding information is as follows: GC was supported by the European Union European Regional Development Fund through the Centre of Excellence in Genomics to Estonian Biocentre and University of Tartu, by Estonian Personal grants PUT-766 and Estonian Institutional Research grants IUT24-1.

The Acknowledgement section is missing from the text. The Acknowledgements are as follows: We thank both of the anonymous reviewers for their constructive criticism. Computational analyses were carried out at the High Performance Computing Center (HPC), University of Tartu and the Core Computing Unit of the Estonian Biocentre.
