# Supplementary figures and images for: Genetic Affinity of the Bhil, Kol and Gond Mentioned in Epic Ramayana
Source: PLoS One. 2015 Jun 10;10(6):e0127655. doi: 10.1371/journal.pone.0127655 (PMC4465503; doi:10.1371/journal.pone.0127655)

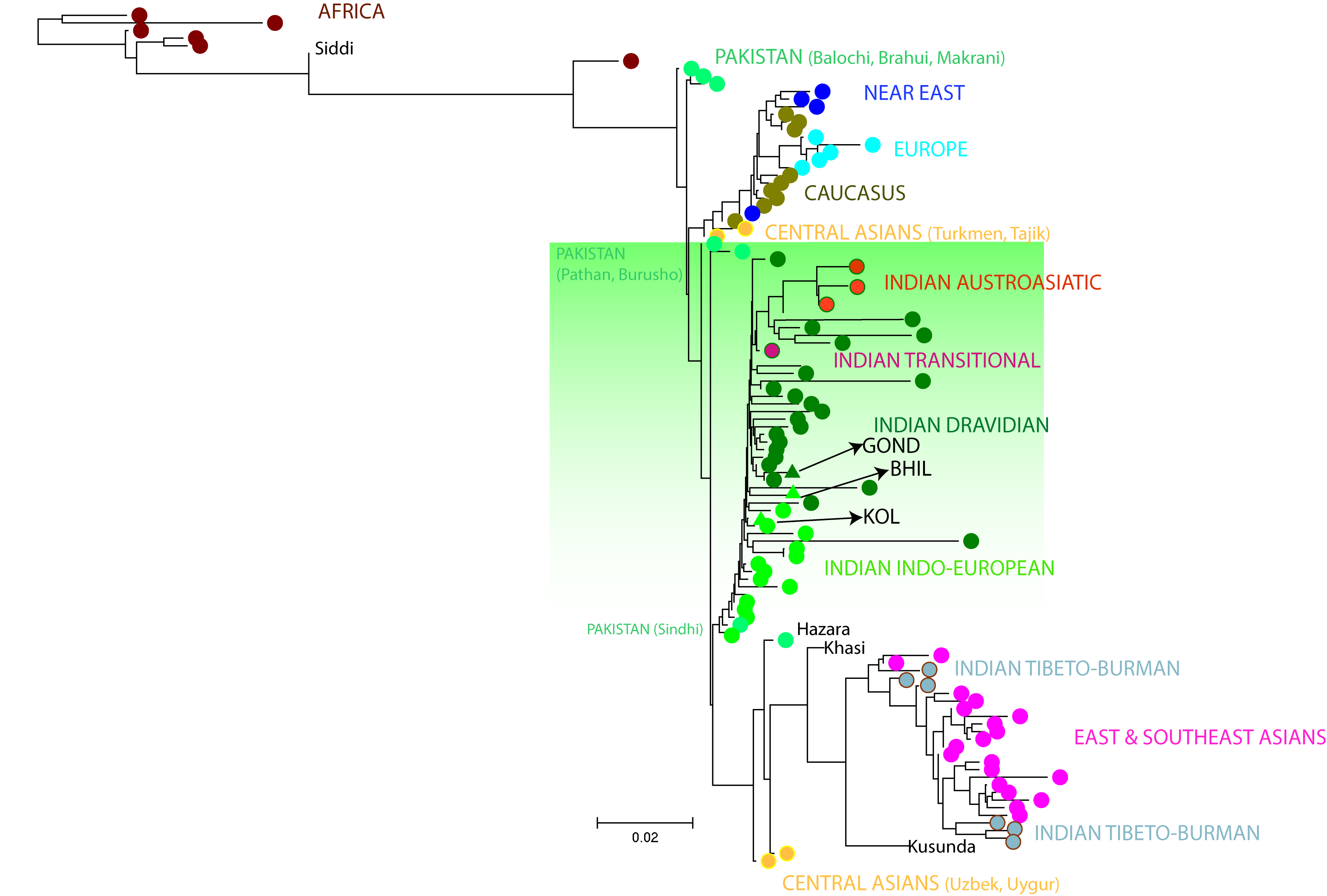

Supplement: S1 Fig — In the inset, the heatmap showing the inter and intra regional genetic affinity of the three tribal populations under investigation. (TIF) [file pone.0127655.s001.tif]

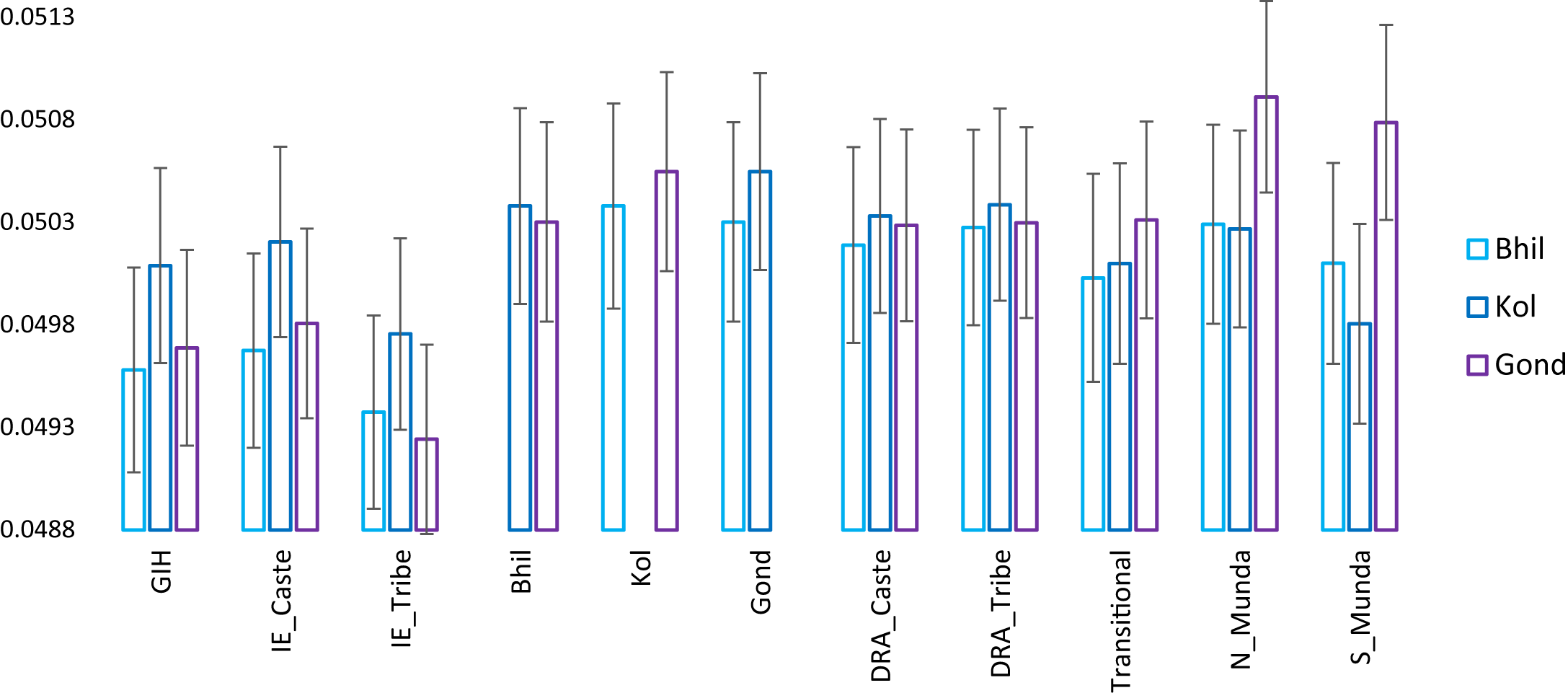

Supplement: S2 Fig — The f3 values are plotted on Y axis against the X- targeted populations on X axis. C_Asia- Central Asia, IN_IE_Caste- Indian Indo-European Caste, IN_IE_Tribe—Indian Indo-European Tribe, IN_DRA_Caste- Indian Dravidian Caste, IN_DRA_Tribe—Indian Dravidian Tribe, IN_AA- Indian Austroasiatic (Munda). (TIF) [file pone.0127655.s002.tif]

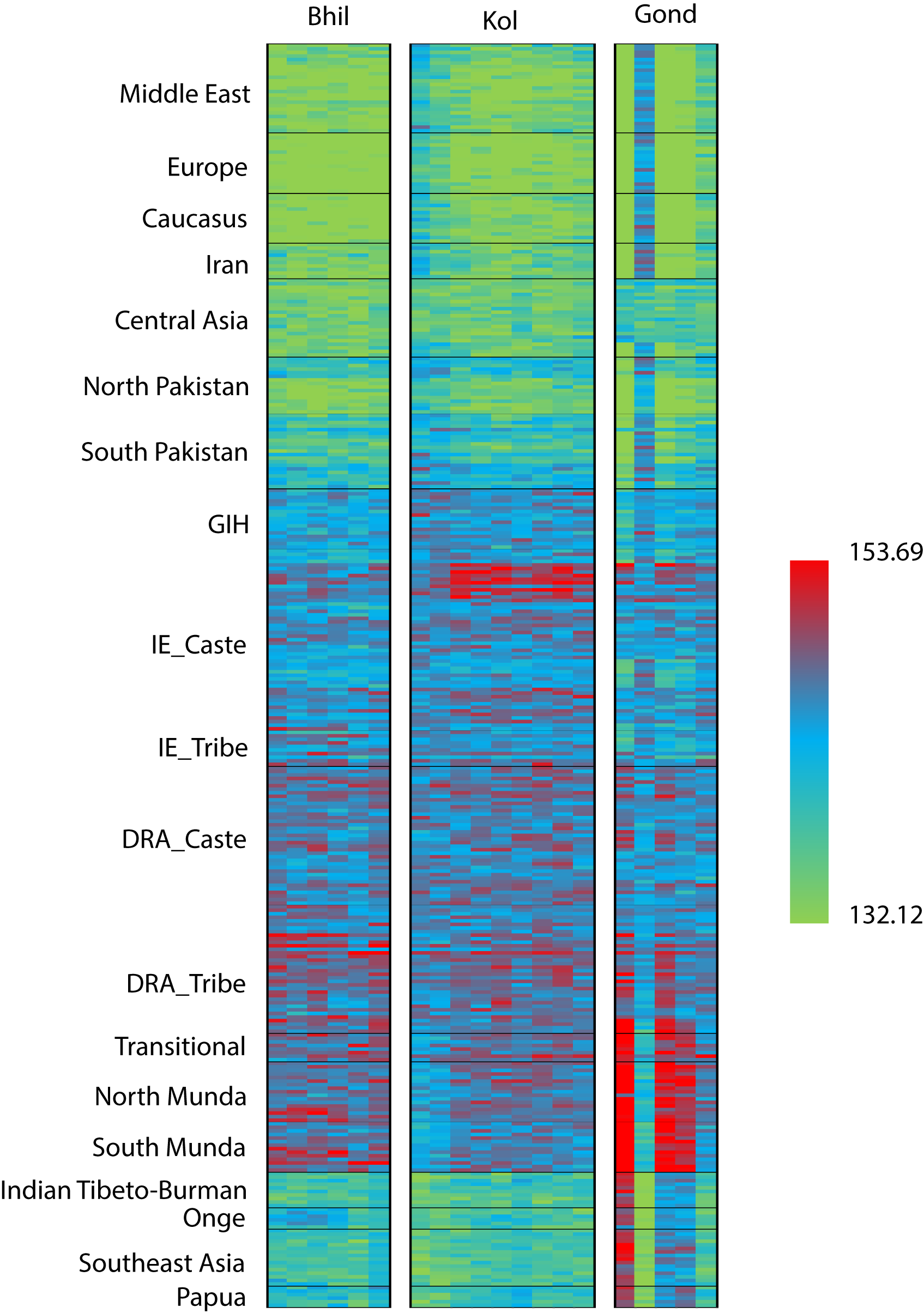

Supplement: S3 Fig — (TIF) [file pone.0127655.s003.tif]

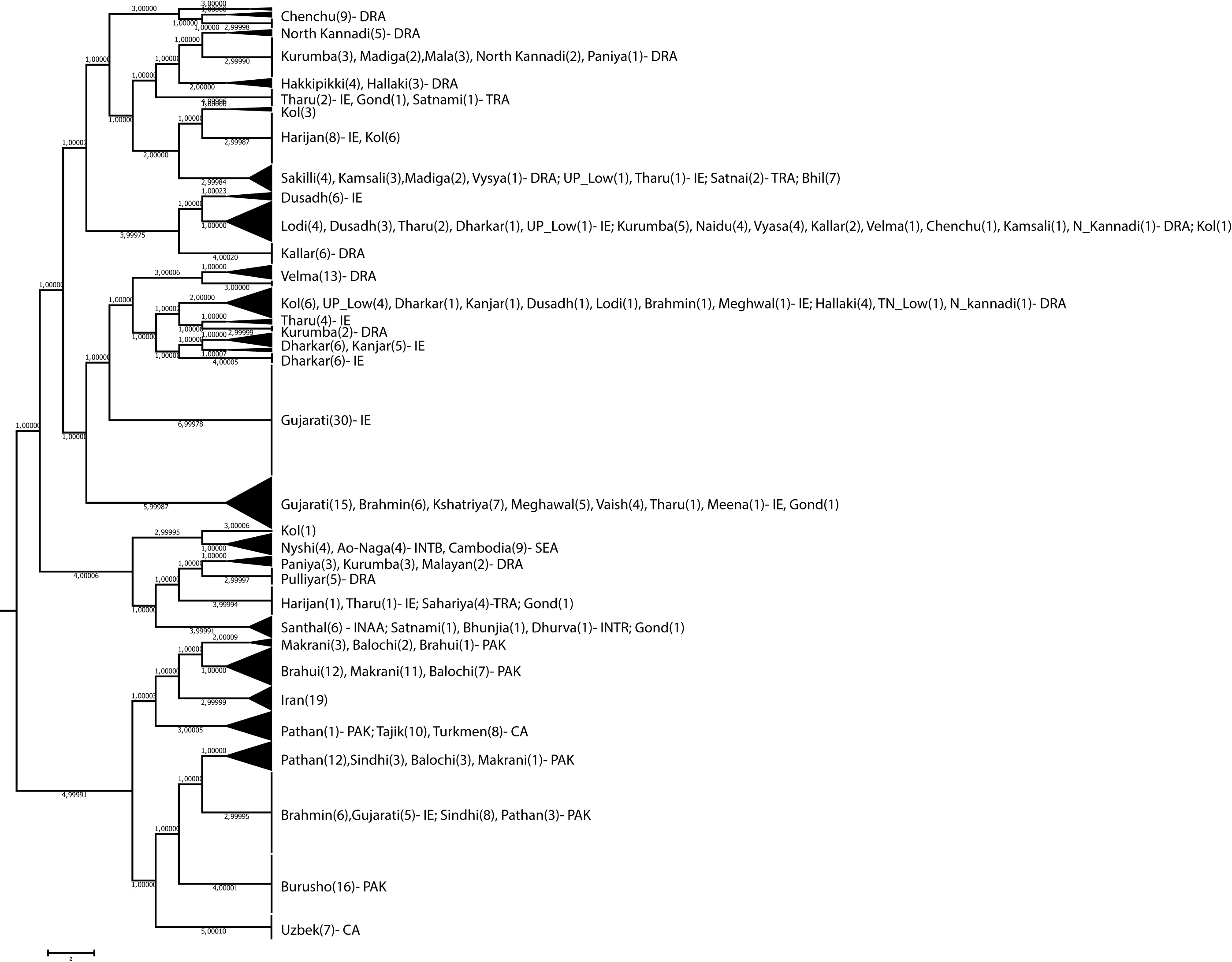

Supplement: S4 Fig — (TIF) [file pone.0127655.s004.tif]
